# O-23 - MULTI-REGION FOLLOW-UP OF A CASE OF LASSA FEVER

**DOI:** 10.1093/pubmed/fdag046.020

**Published:** 2026-07-09

**Authors:** Dr Samihah Moazam, Alethea Charlton, Dr Karthik Paranthaman, Elizabeth Smout, Dr Yimmy Chow, Kerry Cumiskey, Dominic Mellon, Emma Ackermann

**Affiliations:** UK Health Security Agency; UK Health Security Agency; UK Health Security Agency; UK Health Security Agency; UK Health Security Agency; UK Health Security Agency; UK Health Security Agency; UK Health Security Agency

## Abstract

**Background:**

Lassa virus is an important cause of severe viral haemorrhagic fever. It is usually acquired from rodent excreta, but it is also transmissible between humans, particularly through bodily fluids. In March 2025, the UK International Health Regulations National Focal Point (NFP) was informed by the Nigerian NFP of a confirmed case of Lassa fever who had travelled whilst symptomatic from Nigeria to the UK in late February 2025. The case had died in Nigeria. The primary objective was to rapidly identify contacts in the UK and provide public health advice.

**Methods:**

Following notification, contact tracing activities commenced. Investigations revealed the case had travelled to four different regions in England and used several modes of both public and private transport. Data on details of contacts and follow-up status were recorded in a shared line list. Following identification of numerous low-risk exposures, Lassa fever contact tracing matrix and contact information sheets were reviewed and updated.

**Results:**

A total of 127 contacts were identified, 25 of which were non-UK nationals and followed up via the UK NFP. Of the remaining 102 individuals, five were unable to be followed up, and the remaining 97 completed their 21-days post-contact period. Contacts were managed according to the revised guidance, which included active monitoring for the highest risk contacts and providing the appropriate advice. Six contacts were tested; all test results were returned as negative. There were no secondary cases identified.

**Conclusions:**

Contact tracing was critical in confirming the absence of secondary cases and providing appropriate public health advice to exposed persons. Coordination of activities among the teams across UKHSA, including a collated line list of exposed persons and provision of mutual aid, was paramount to ensuring a rapid and effective response. Several important lessons were identified in the debrief exercise.

